# Identification of Factors for the Preoperative Prediction of Tumour Subtype and Prognosis in Patients with T1 Lung Adenocarcinoma

**DOI:** 10.1155/2016/9354680

**Published:** 2016-12-26

**Authors:** Wang-Yu Zhu, Yong-Kui Zhang, Zhen-da Chai, Xiao-fei Hu, Lin-lin Tan, Zhao-yu Wang, Zhi-jun Chen, Han-bo Le

**Affiliations:** ^1^Laboratory of Cytobiology and Molecular Biology, Zhoushan Hospital of Wenzhou Medical University, Zhoushan, Zhejiang 316021, China; ^2^Lung Cancer Research Centre, Zhoushan Hospital of Wenzhou Medical University, Zhoushan, Zhejiang 316021, China; ^3^Department of Cardio-Thoracic Surgery, Zhoushan Hospital of Wenzhou Medical University, Zhoushan, Zhejiang 316021, China; ^4^Department of Science Education, Zhoushan Hospital of Wenzhou Medical University, Zhoushan, Zhejiang 316021, China

## Abstract

*Aims.* Identification of factors that can predict the subtypes of lung adenocarcinoma preoperatively is important for selecting the appropriate surgical procedure and for predicting postoperative survival.* Methods.* We retrospectively evaluated 87 patients with lung adenocarcinomas ≤30 mm.* Results.* Preoperative radiological findings, serum CEA level, serum microRNA-183 (miR-183) level, and tumour size differed significantly between patients with adenocarcinoma in situ (AIS) or minimally invasive adenocarcinoma (MIA) and those with invasive adenocarcinoma (IAC). Receiver operating characteristic curves and univariate analysis revealed that patients who were older than 57 years or had a pure solid nodule or a tumour with mixed ground-glass opacity (mGGO), a tumour >11 mm, a serum CEA level >2.12 ng/mL, or a serum miR-183 level >1.233 (2^−ΔΔCt^) were more likely to be diagnosed with IAC than with AIS or MIA. The combination of all five factors had an area under the curve of 0.946, with a sensitivity of 89.13% and a specificity of 95.12%. Moreover, patients with a cut-off value >0.499 for the five-factor combination had poor overall survival.* Conclusions.* The five-factor combination enables clinicians to distinguish AIS or MIA from IAC, thereby aiding in selecting the appropriate treatment, and to predict the prognosis of lung adenocarcinoma patients.

## 1. Introduction

The widespread use of computed tomography (CT) for screening lung cancers has made the detection of small peripheral pulmonary nodules possible [1]. Limited surgical resection has gradually gained acceptance for the treatment of such nodules and has excellent outcomes superior to those of lobectomy [2]. The most common histological type of lung cancer in recent years is adenocarcinoma [3]. The favourable prognosis of tumours containing larger areas of GGO appears to be independent of the tumour subtype and thus is potentially treatable via limited surgical resection [4–6]. However, because of the high frequency of lymph node involvement, use of this procedure is still controversial. Moreover, tumours <3 cm with both GGO areas and solid areas are often aggressive and invasive [7]. The lung adenocarcinoma subtypes have distinct GGO patterns [8] and thus can be readily identified via imaging; identification of the subtype is essential for selecting the appropriate surgical procedure for patients with small-sized lung cancers. Serum carcinoembryonic antigen (CEA) is also a useful diagnostic and prognostic factor for patients with lung cancer [9, 10]. Tomita et al. reported that lung adenocarcinomas with the nonlepidic dominant histologic subtype typically have high serum levels of CEA [11].

A better understanding of the molecular biology of lung adenocarcinoma might allow surgeons to better predict patient outcomes, as well as to define its subtypes. Our studies identified circulating microRNAs (miRNAs) as potential diagnostic biomarkers for early-stage lung cancer and suggest that several miRNAs can distinguish lung adenocarcinomas from squamous cell lung cancers [9, 12–14]. To our knowledge, only a few studies have shown that miRNAs can do so. Previous studies found that** s**erum levels of the miRNA, miR-183, were higher in patients with lung cancer than in healthy individuals and that miR-183 promoted tumour cell growth and migration (i.e., acted as onco-miRNA) by targeting the transcription factor early growth response protein 1 [12, 15–17].

The combination of mRNA expression, miRNA expression, and DNA methylation has been used to identify prognostic classifiers for lung adenocarcinoma [18]; however, to our knowledge, no studies have evaluated the usefulness of combined CT findings, CEA levels, and miRNA expression. The aim of this study was to determine whether these parameters preoperatively predict prognosis, postoperative histological subtype, and lymph node involvement in patients with lung adenocarcinoma, toward the overall goal of identifying the subgroups that could benefit from limited resection. Our study was performed on patients with resected lung adenocarcinomas <30 mm.

## 2. Materials and Methods

### 2.1. Study Population

We retrospectively reviewed and analysed 87 consecutive patients (38 men, 49 women; mean age, 58 years; age range, 27–81 years) with pathological T1 lung adenocarcinoma (tumour size < 30 mm) who underwent surgical resection with curative intent at the Zhoushan Hospital (Zhejiang, China) between December 2011 and September 2014 and who were monitored for local recurrence and distant metastasis in follow-ups. Before surgery, all patients underwent routine or contrast-enhanced chest CT (Sensation 16; Siemens, Erlangen, Germany). The patients also underwent preoperative cardiopulmonary tests, abdominal CT or abdominal ultrasonography, brain magnetic resonance imaging or brain CT, and bone scanning. All patients underwent lobectomy with hilar and mediastinal lymphadenectomy or limited resection (segment or wedge) with lymph node sampling; none received preoperative chemotherapy or radiography. All specimens were formalin-fixed and stained with haematoxylin and eosin after surgery.

Non-small cell lung cancer (NSCLC) was diagnosed histologically by two pathologists in accordance with the 2004 World Health Organization criteria [19]. In accordance with the revised criteria of the International Association for the Study of Lung Cancer/American Thoracic Society/European Respiratory Society (IASLC/ATS/ERS) [3], lung adenocarcinoma was classified as adenocarcinoma in situ (AIS), minimally invasive adenocarcinoma (MIA), or invasive adenocarcinoma (IAC). For IAC, there are five predominant growth patterns (lepidic, acinar, papillary, solid, and micropapillary) and four variants (invasive mucinous, colloid, foetal, and enteric) [20]. We also collected clinicopathological factors, including age, sex, lymphatic and vascular vessel invasion, bronchial invasion, preoperative serum CEA levels, and surgical procedures.

Blood was collected from all patients and 48 healthy individuals (normal controls) early in the morning. The serum was separated immediately after blood collection and stored at −80°C until use. The laboratory technicians were blinded to the patient's identity [17]. This study was approved by the Ethical Review Committee of the Zhoushan Municipal Government of China, and all patients provided written informed consent.

### 2.2. CT Imaging

**Figure 1:**
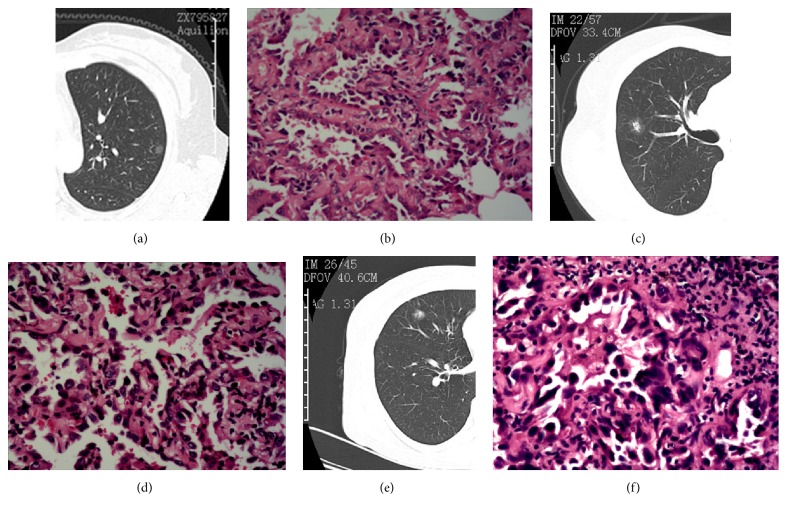
Radiological and histopathological findings for the different subtypes of lung adenocarcinoma. (a) High-resolution computed tomography (HRCT) scan of an adenocarcinoma in situ 8 mm in diameter with a radiologically pure ground-glass opacity (GGO) nodule. (b) Microscopic examination of the tumour in (a). (c) HRCT scan of a minimally invasive adenocarcinoma 16 mm in diameter with a radiologically mixed GGO nodule. (d) Microscopic examination of the tumour in (c). (e) HRCT scan of a papillary-predominant invasive adenocarcinoma 13 mm in diameter with a radiologically mixed GGO nodule. (f) Microscopic examination of the tumour in (e). All photographs show haematoxylin-eosin staining; original magnification ×400.

High-resolution CT (HRCT) images were acquired by using a 16-row multislice CT scanner and the following parameters: 120 kVp and autoexposure control, 140–170 mA, 0.75-mm slice collimation, 12-mm feed/rotation, 0.5-second rotation time, and lung window settings (level = −500/width = 1500 HU). Moreover, all tumours were subsequently evaluated to estimate the extent of GGO via image reconstruction using a 1.0 mm slice thickness and a lung algorithm. In the current study, GGO was defined as the presence of misty increases in lung attenuation that did not obscure the bronchial or vascular walls, whereas solid tumours were tentatively defined as tumours in which the maximum diameter of consolidation relative to the maximum diameter of the tumour (consolidation/total tumour size) was >5 mm. Based on the diameters determined via HRCT, tumours were further classified into three groups: pure GGO (pGGO) (tumours without a solid component), mGGO (tumours with both GGO and solid components), and pure solid nodules (tumours with a solid component only) (Figure 1).

### 2.3. Serum Preparation, RNA Isolation, and Quantitative Reverse Transcription-Polymerase Chain Reaction

To obtain serum samples, 10 mL aliquots of peripheral blood were drawn into separate gel tubes and centrifuged within 30 min at 1,500 ×g for 10 min at 4°C. The supernatants were transferred to 1.5 mL tubes and stored at −80°C until use. For detection of miRNA, RNA was isolated from 600 *μ*L serum samples using a mirVana PARIS RNA isolation kit (Applied Biosystems, Foster City, CA, USA) as specified by the manufacturer. RNA concentration was determined by using a NanoDrop ND-1000 spectrophotometer (NanoDrop Technologies, Wilmington, DE, USA). Reverse transcription reactions were performed by using a TaqMan MicroRNA Reverse Transcription Kit (Applied Biosystems) according to the manufacturer's instructions. Quantitative reverse transcription-polymerase chain reaction (qRT-PCR) was subsequently carried out on triplicate serum samples using TaqMan 2x Universal PCR Master Mix with no AmpErase UNG (Applied Biosystems) and an ABI 7500 Real-Time PCR system (Applied Biosystems). The qRT-PCR amplification conditions consisted of an initial cycle at 95°C for 10 min, followed by 40 cycles at 95°C for 15 s and 60°C for 1 min. Cycle threshold (Ct) values were calculated by using SDS 2.0.1 software (Applied Biosystems). No template controls were used at either the RT or PCR steps to ensure target-specific amplification. The control miRNA was U6 snRNA [21]. The 2^−ΔΔCt^ method was used to calculate the average levels of serum miRNA (which were relative to the average levels of U6 snRNA) and to determine the fold change in the expression of the target miRNA relative to that of the miRNAs expressed in normal controls [22]. The mean Ct values, excluding outliers (i.e., replicates with Ct values differing by more than one cycle from the median), for miR-183 were calculated. In addition, if the average U6 Ct was not within 20 or 32 cycles, the assay was repeated at least once on some of the samples. Samples with low U6 snRNA levels were not included in the data analysis [17].

### 2.4. Follow-Up of the Patients

All patients were followed-up in our outpatient clinic at 3-month intervals for the first year and 6-month intervals thereafter. Tumour recurrence was assessed via CT and by measuring serum CEA levels. The length of the follow-up period ranged from 2 to 46 months, with a mean of 29 months and a median of 32 months. The last follow-up was conducted in June 2015. The endpoint of this study was the death of the patient or was censored at the time of the last follow-up. Overall survival was defined as the time (in months) from surgery to either death or the last follow-up.

### 2.5. Statistical Analysis

Statistical analyses were performed by using GraphPad Prism 5.0 software (GraphPad Software Inc., San Diego, CA, USA) and MedCalc 9.0 software (MedCalc Software Inc., Mariakerke, Belgium). The data were examined according to the degree of homogeneity. The unpaired* t-*test or Mann–Whitney* U *test was used to analyse the differences between patients and controls and the correlations between miRNA expression levels and the clinicopathological features of the patients. Receiver operating characteristic (ROC) curves were generated to assess the diagnostic accuracy of each parameter. The areas under the curves (AUCs) were calculated and compared with each other via a nonparametric approach.

The five factors (age, serum CEA level, serum miR-183 level, tumour size, and nodule type) that differed significantly between patients with AIS or MIA and those with IAC were combined to predict AIS and MIA versus IAC; multiple logistic regression analysis and MedCalc 9.0 software were used to calculate the predictive value. Multiple logistic regression analysis was also performed to assess the diagnostic accuracy of total serum miRNA levels in the lung adenocarcinoma patients. All statistical tests were two-sided, and a* p *value ≤ 0.05 was considered statistically significant.

## 3. Results

The characteristics of the patients and tumours are summarized in Table 1. The tumours were stratified into two groups according to their pathological characteristics: AIS or MIA (41 patients, 47.1%) and IAC (46 patients, 52.9%) (Figure 1). Neither lymph node metastasis nor visceral pleural invasion was observed in patients with AIS, MIA, or lepidic-predominant IAC. Of the four patients with lymph node metastasis, three had papillary-predominant IAC and one had solid-predominant IAC. Pure solid nodules were also not observed in the patients with AIS or MIA. One of the 23 patients with papillary-predominant IAC presented with pGGO. Preoperative radiological findings, serum levels of CEA, serum levels of miR-183, tumour size, and visceral pleural invasion status differed significantly between patients with AIS or MIA and those with IAC (*p*: <0.001, 0.015, 0.042, <0.001, and 0.003, resp.) (Table 2). Median age was higher in the IAC group than in the AIS and MIA group (*p* < 0.001).

**Figure 2:**
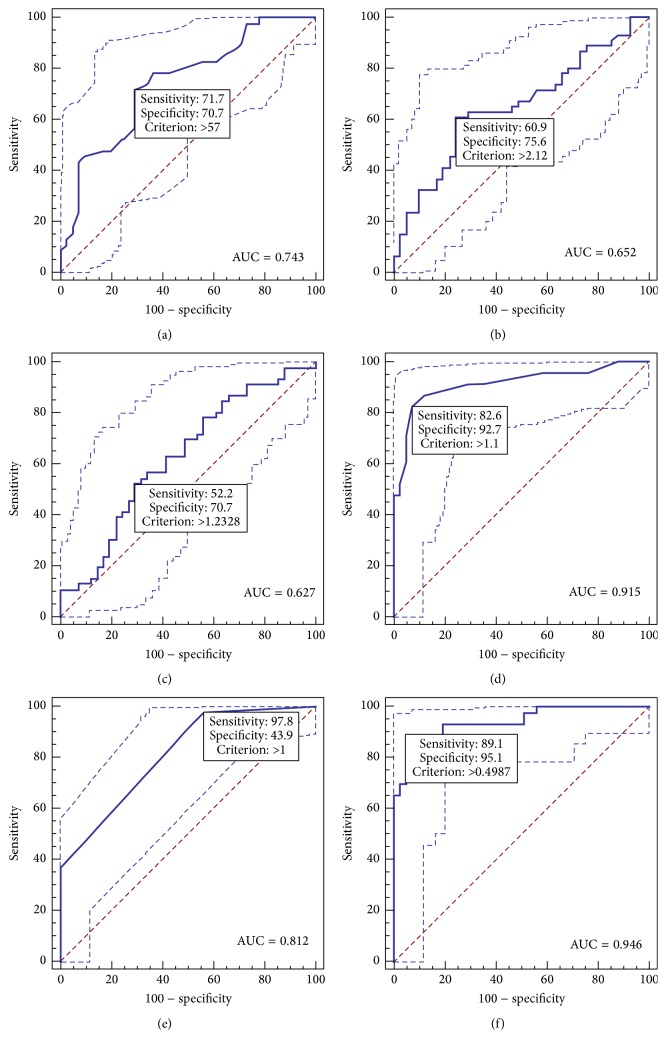
Receiver operating characteristic curves assessing the accuracy of several factors in predicting invasive adenocarcinoma (IAC) (41 patients) versus adenocarcinoma in situ or minimally invasive adenocarcinoma (41 patients). The *p* values for age (a), serum carcinoembryonic antigen (CEA) level (b), microRNA-183 (miR-183) level (c), tumour size (d), nodule type (e), and the combination of these five factors (f) as determined via logistic regression were <0.001, 0.010, 0.035, <0.0001, <0.001, and <0.001, respectively. AUC, area under the curve; pGGO, pure ground-glass opacity; mGGO, mixed ground-glass opacity.

Factors that predict the IAC subtype versus the AIS and MIA subtypes were identified by using a logistic regression model. In the univariate analysis, older age, the presence of pure solid nodules, a high level of CEA, and tumour size >20 mm significantly predicted the IAC subtype (*p*: 0.001, <0.001, 0.024, and <0.001, resp.) (Table 3). In the multivariate analysis, the presence of pure solid nodules and tumour size >20 mm were independent predictors of the IAC subtype (*p*: 0.017 and <0.001, resp.). ROC curves were generated to assess the IAC prediction accuracy of the four factors identified in the univariate analysis, as well as miR-183.  Figure 2 shows the true-positive ratios (sensitivity) and false-positive ratios (1 minus specificity) for these factors. The AUCs for age, serum CEA, miR-183, tumour size, and nodule type were, respectively, 0.743, 0.652, 0.627, 0.915, and 0.812 (*p* < 0.001), with a sensitivity of 71.74%, 60.87%, 52.17%, 82.61%, and 97.83% (*p* = 0.001) and a specificity of 70.73%, 75.61%, 70.73%, 92.68%, and 43.90% (*p* < 0.001). We also assessed the IAC prediction accuracy of these five factors in combination via multivariate logistic regression analysis. The AUC of the five-factor combination was 0.946, with a sensitivity of 89.13% and a specificity of 95.12% (*p* < 0.001).

To further distinguish IAC from AIS and MIA, we performed univariate and multivariate analyses using the optimal cut-off values calculated from the ROC curves. These were 57 years, 2.12 ng/mL, 1.233 (2^−ΔΔCt^), 11 mm, 1, and 0.499 for age, serum CEA, miR-183, tumour size, pGGO or mGGO versus pure solid nodules, and the five-factor combination, respectively (Table 4). According to the univariate analysis, patients were more likely to be diagnosed with IAC than with AIS or MIA if they were >57 years of age or had a pure solid nodule, a tumour size >11 mm, a serum CEA level >2.12 ng/mL, or a miR-183 level >1.233 (2^−ΔΔCt^) (*p*: <0.001, 0.001, <0.001, 0.001, and 0.020, resp.). Using the cut-off value of 0.499, the combination of these five factors also indicated a high possibility of IAC and was an independent diagnostic factor for IAC in the multivariate analysis (*p* < 0.001).

**Figure 3:**
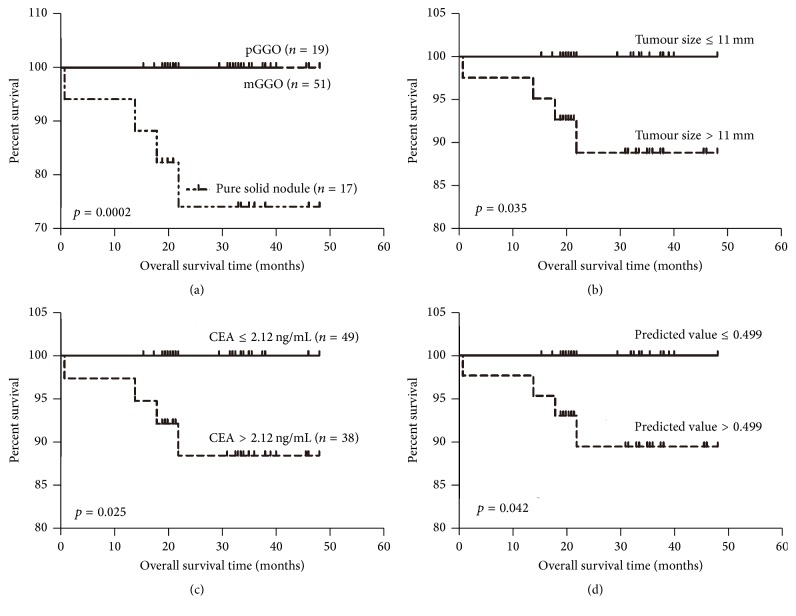
Kaplan-Meier analysis of overall survival in patients with lung adenocarcinoma. Overall survival was stratified according to nodule type (a) and the optimal cut-off values for tumour size (b), serum carcinoembryonic antigen (CEA) level (c), and the five-factor combination (d).

Additional studies assessed the overall survival of patients with lung adenocarcinoma according to the preoperative factors identified above using the optimal cut-off values. The results of these studies showed that patients with >1 pure solid nodule, a tumour >11 mm, a serum CEA level >2.12 ng/mL, or a predictive value >0.499 had shorter overall survival times than their respective counterparts (*p*: 0.0002, 0.035, 0.025, and 0.042, resp.) (Figure 3). However, there were no significant differences between patients >57 and ≤57 years of age (*p* = 0.874) or with a serum miR-183 level >1.233 and ≤1.233 (2^−ΔΔCt^) (*p* = 0.854).

## 4. Discussion

This study shows that specific preoperative factors can predict the pathological subtype of T1 lung adenocarcinomas. The five-factor combination of age, nodule type, serum CEA level, serum miR-183 level, and tumour size had an AUC of 0.946, with a sensitivity of 89.13% and a specificity of 95.12%. Moreover, based on optimal cut-off values, a tumour size >11 mm and a serum CEA level >2.12 ng/mL predicted the prognosis of lung adenocarcinoma patients, as did the presence of a pure solid nodule and a value >0.499 for the five-factor combination.

The IASLC/ATS/ERS classification system clearly presents the prognosis values for the predominant pathological subtypes and categorizes tumour behaviour according to the genetic diversity of the subtypes [23–25]. Two studies showed that lung adenocarcinoma patients with AIS or MIA but no lymph node metastasis had a nearly 100% overall survival rate, whereas those with histologic acinar-, papillary-, solid-, or micropapillary-predominant IAC had relatively poor prognosis [25, 26]. Limited resection has been recently advocated for patients with AIS or MIA because it preserves lung function [27] but is not suitable for patients with IAC. Thus, using preoperative factors to predict the histologic subtype of lung adenocarcinomas would be helpful for determining the appropriate surgical procedure.

Because preoperative diagnosis of AIS and MIA via transbronchial biopsy or percutaneous needle biopsy is difficult, measurement of circulating tumour marker levels and CT imaging, which is a noninvasive procedure, are attractive alternatives. Image analysis via high-resolution chest CT provides the most useful information for identifying AIS and MIA [6]. Our results showed that pGGO was closely associated with AIS and MIA in most instances (as the exception, one patient with a pGGO tumour presented with papillary-predominant IAC). In a recent study, only 12% of cases with pGGO were ultimately diagnosed as IAC, most of which were papillary-predominant [28]. In our study, pure solid nodules were associated with IAC but not with AIS or MIA, and there was no obvious association of mGGO with AIS, MIA, or IAC. The presence of a pure solid nodule independently predicted IAC with an AUC of 0.812, and patients with a pure solid nodule had a worse survival rate than did those with a pGGO or mGGO tumour. In agreement with our results, several studies found that the proportion of GGO areas relative to the total tumour area strongly predicted overall survival in patients with clinical T1N0M0 lung adenocarcinoma [29, 30]. Although our study shows that GGO is useful for diagnosing and predicting the prognosis of the adenocarcinoma subtypes, other factors should be identified to improve sensitivity and specificity, especially for mGGO tumours owing to their high recurrence rate [29].

The proposed eighth edition of the tumour, node, and metastasis (TNM) classification system for lung cancer suggests revisions that might improve lung cancer diagnosis and potentially subcategorize T1 tumours as T1a (≤10 mm), T1b (>10 to ≤20 mm), or T1c (>20 to ≤30 mm) [31]. We found that tumour size was an independent diagnostic factor for IAC that remarkably distinguished IAC from AIS and MIA when an optimal cut-off value of 11 mm was used. Mao et al. reported that 8.9 mm was the optimal tumour size cut-off value for differentiating between preinvasive (atypical adenomatous hyperplasia and AIS) and invasive (MIA and IAC) lesions [32]. However, Lee et al. recommended optimal cut-off values of 10 mm and 14 mm for distinguishing preinvasive lesions from invasive pulmonary lesions in cases of pGGO and partly sGGO, respectively [33]. The appropriate value might differ according to the size of the lung window and patient cohort, which would be determined by the institute. Larger tumour size significantly correlated with a higher percentage of metastatic N2 lymph nodes and poor prognosis in patients with T1 lung adenocarcinoma [34]. Our results also demonstrated that a tumour size >11 mm predicted poor overall survival. Further surgical intervention and radiotherapy or chemotherapy should be indicated for such tumours.

Serum CEA is a useful circulating biomarker and prognostic factor for lung cancer. However, the optimal cut-off value for serum CEA level varies in the literature [7, 9]. Using the optimal cut-off value identified in our study (>2.12 ng/mL), we found that serum CEA level was associated not only with IAC, but also with poor overall survival in T1 lung adenocarcinoma patients [11, 35]. Several studies have shown a correlation between high preoperative serum CEA levels and poor survival after lung cancer resection, which is consistent with our results [7, 11, 36].

Circulating miRNAs were identified as novel, noninvasively detected biomarkers for diagnosing and predicting the prognosis of lung cancers [9, 12, 37, 38]. Herein, we are the first to report that elevated levels of the onco-miRNA, miR-183, were closely associated with IAC and could distinguish IAC from AIS and MIA when an optimal cut-off value of 1.2328 (AUC = 0.627) was used. Previous studies showed increased circulating levels of miR-183 in several cancer types and suggested that miR-183 was a potential biomarker for the diagnosis of carcinomas including lung carcinomas [12, 17, 39, 40].

Nevertheless, it was sometimes difficult to accurately predict the lung adenocarcinoma subtype and thus the surgical procedure that would be most successful, using only a single factor. To potentially improve accuracy, we combined the five factors (age, nodule type, serum CEA level, serum miR-183 level, and tumour size) that significantly differentiated AIS and MIA from IAC preoperatively. This resulted in a cut-off value (>0.499) that further increased diagnostic accuracy (AUC, 0.946; 89.1% sensitivity, and 95.1% specificity) and adequately excluded IAC. The five-factor combination represents a useful means of selecting patients suitable for limited resection, and it was remarkably associated with poor prognosis in our study. Therefore, we successfully validated the use of limited resection by using the following parameters: age ≤57 years, serum CEA level ≤2.12 ng/mL, serum miR-183 level ≤1.233 (2^−ΔΔCt^), tumour size ≤11 mm, and the absence of a pure solid nodule.

In conclusion, our study highlights the potential usefulness of radiological findings, serum CEA and miR-183 levels, and tumour size in predicting the subtype and prognosis of lung adenocarcinomas. The five-factor combination allows clinicians to distinguish AIS and MIA from IAC and to predict the prognosis of lung adenocarcinomas for which limited surgical resection or adjusted therapy is appropriate. Further studies with more patients and longer follow-up periods after limited resection are recommended.

## Figures and Tables

**Table 1:** Characteristics of the patients and tumours.

Variable	Category	Number (%)
Sex	Male	38 (43.7)
	Female	49 (56.3)

Median age (range)		58.0 ± 11.1 (27–81)

Smoking	Current smoker	66 (75.9)
	Never smoked	21 (24.1)

Radiological finding	Pure GGO	19 (21.8)
	Mixed GGO	51 (58.6)
	Pure solid nodule	17 (19.6)

CEA	≤5 ng/mL	74 (85.1)
	>5 ng/mL	13 (14.9)

Serum miR-183	≤1.0 (2^−ΔΔCt^)	43 (49.4)
	>1.0 (2^−ΔΔCt^)	44 (50.6)

Tumour size	≤10 mm	42 (48.3)
	>10 to ≤20 mm	30 (34.5)
	>21 to ≤30 mm	15 (17.2)

Nodule number	Single	82 (94.3)
	Multiple	5 (5.7)

Predominant subtype	AIS	19 (21.8)
	MIA	22 (25.3)
	Lepidic-predominant IAC	11 (12.7)
	Papillary-predominant IAC	23 (26.4)
	Acinar-predominant IAC	9 (10.3)
	Solid-predominant IAC	2 (2.3)
	Invasive mucinous IAC	1 (1.2)

Lymph node metastasis	Present	4 (4.6)

Visceral pleural invasion	Present	9 (10.3)

Pathological stage	0	5 (5.7)
	I	76 (87.4)
	II	4 (4.6)
	III	2 (2.3)

CEA, carcinoembryonic antigen; GGO, ground-glass opacity; AIS, adenocarcinoma in situ; MIA, minimally invasive adenocarcinoma; IAC, invasive adenocarcinoma.

**Table 2:** Correlation of the resected lung adenocarcinoma classification (*n* = 87) with clinical characteristics.

Variable	Category	AIS and MIA (*n* = 41)	IAC (*n* = 46)	*P *value
Sex	Male	15 (36.6)	23 (50.0)	0.279
	Female	26 (63.4)	23 (50.0)	

Median age (years)		52.8 ± 10.3	62.7 ± 9.8	<0.001^*∗*^

Smoking	Always smoked	33 (80.5)	33 (71.7)	0.453
	Never smoked	8 (19.5)	13 (28.3)	

Radiological finding	Pure GGO	18 (43.9)	1 (2.2)	<0.001^*∗*^
	Mixed GGO	23 (56.1)	28 (60.9)	
	Pure solid nodule	0	17 (36.9)	

CEA [IQR (median)] (ng/mL)		1.17–2.32 (1.78)	1.48–4.31 (2.30)	0.015^*∗*^

Serum miR-183 [IQR (median)]		0.232–1.63 (0.790)	0.684–4.33 (1.325)	0.042^*∗*^

Tumour size	≤10 mm	36 (87.8)	6 (13.0)	<0.001^*∗*^
	>10 to ≤20 mm	4 (9.8)	26 (56.5)	
	>21 to ≤30 mm	1 (2.4)	14 (30.5)	

Lymph node metastasis	Absent	41 (100.0)	42 (91.3)	0.119
	Present	0	4 (8.7)	

Visceral pleural invasion	Absent	41 (100.0)	37 (80.4)	0.003^*∗*^
	Present	0	9 (19.6)	

Pathological stage	0/IA	40 (97.6)	30 (65.2)	<0.001^*∗*^
	IB/IIA/IIB/IIIA	1 (2.4)	16 (34.8)	

^*∗*^Statistically significant *p *value

CEA, carcinoembryonic antigen; GGO, ground-glass opacity; IQR, interquartile range; AIS, adenocarcinoma in situ; MIA, minimally invasive adenocarcinoma; IAC, invasive adenocarcinoma.

**Table 3:** Univariate and multivariate analysis to predict pathological subtypes.

Variable	Univariate			Multivariate		
	OR	95% CI	*p* value	OR	95% CI	*p* value
Age >60 years	4.994	2.005–12.438	0.001^*∗*^	2.167	0.547–8.593	0.271
Sex: male	0.577	0.244–1.362	0.209	—	—	—
Smoking status: current	1.625	0.595–4.436	0.343	—	—	—
Pure solid nodule on radiology	36.561	4.972–268.878	<0.001^*∗*^	12.681	1.562–102.972	0.017^*∗*^
Serum CEA level >5 ng/mL	6.129	1.270–29.583	0.024^*∗*^	1.143	0.117–11.131	0.908
Serum miR-183 level >1.0 (2^−ΔΔCt^)	2.006	0.853–4.716	0.110	—	—	—
Tumour size >21 to ≤30 mm	21.981	6.988–69.142	<0.001^*∗*^	9.609	2.882–32.038	<0.001^*∗*^

^*∗*^Statistically significant *p* value; —, not included in the multivariate analysis

OR, odds ratio; CEA, carcinoembryonic antigen; CI, confidence interval.

**Table 4:** Univariate and multivariate analysis to predict pathological subtype using optimal cut-off values.

Variable	Cut-off value	Univariate			Multivariate		
		OR	95% CI	*P *value	OR	95% CI	*P *value
Age	>57 years	6.135	2.421–15.545	<0.001^*∗*^	1.307	0.209–8.175	0.774
Radiological findings	>1 pure solid nodule	35.217	4.420–280.601	0.001^*∗*^	3.304	0.317–34.440	0.318
Serum CEA level	>2.12 ng/mL	4.822	1.909–12.181	0.001^*∗*^	0.957	0.158–5.788	0.961
Serum miR-183 level	>1.233 (2^−ΔΔCt^)	2.900	1.183–7.112	0.020^*∗*^	0.428	0.046–4.006	0.457
Tumour size	>11 mm	60.167	14.822–244.236	<0.001^*∗*^	3.106	0.253–38.068	0.375
Combined five factors	>0.499	159.900	29.287–873.024	<0.001^*∗*^	60.033	2.462–1463.959	0.012^*∗*^

^*∗*^Statistically significant *p* value

OR, odds ratio; CI, confidence interval; mGGO, mixed ground-glass opacity; CEA, carcinoembryonic antigen.
